# Optimal Perceived Timing: Integrating Sensory Information with Dynamically Updated Expectations

**DOI:** 10.1038/srep28563

**Published:** 2016-07-07

**Authors:** Massimiliano Di Luca, Darren Rhodes

**Affiliations:** 1 Centre for Computational Neuroscience and Cognitive Robotics, School of Psychology, University of Birmingham, Edgbaston, Birmingham B15 2TT, UK

## Abstract

The environment has a temporal structure, and knowing when a stimulus will appear translates into increased perceptual performance. Here we investigated how the human brain exploits temporal regularity in stimulus sequences for perception. We find that the timing of stimuli that occasionally deviate from a regularly paced sequence is perceptually distorted. Stimuli presented *earlier* than expected are perceptually delayed, whereas stimuli presented *on time* and *later* than expected are perceptually accelerated. This result suggests that the brain regularizes slightly deviant stimuli with an asymmetry that leads to the perceptual acceleration of expected stimuli. We present a Bayesian model for the combination of dynamically-updated expectations, in the form of *a priori* probability of encountering future stimuli, with incoming sensory information. The asymmetries in the results are accounted for by the asymmetries in the distributions involved in the computational process.

Events in our perceptual world often have a predictable temporal structure. Exploiting temporal regularities can decrease metabolic consumption^1^ and automatize behavior for rhythmic activities such as dance, locomotion, speech, and music production^2^,^3^. Predictable timing of events leads to improved stimulus detection and discrimination^4^,^5^,^6^,^7^,^8^,^9^,^10^,^11^, perceptual changes^12^, and faster responses^7^,^8^,^13^,^14^. The computational mechanisms behind these perceptual phenomena are unclear. Here we propose to use one of the simplest types of stimulus regularities, the occurrence of stimuli after equal intervals of time (*isochrony*), to quickly generate the expectation for a successive stimulus. We investigate how perceived timing changes due to such an expectation.

There are several schools of thought about how the brain deals with the regularity of stimulus sequences. *Interval-based models* assert that the time between two stimuli is represented as a discrete interval duration that is compared with subsequent intervals^15^,^16^,^17^,^18^. The representation is refined when more stimuli are presented, leading to increased performance^19^,^20^,^21^,^22^. In contrast, *entrainment models* advocate that the phase and frequency of temporal patterns is the important aspect. The dynamics of attending to stimuli, for example, has been shown to adjust to rhythmic external stimulation^23^,^24^,^25^,^26^. At a neural level, phase coincidence^27^ and activity patterns^28^,^29^ progressively tune to the phase and frequency of rhythmic stimulus sequences. Exogenous attention is then deployed at the expected time^30^,^31^. Interestingly, attention and expectation have opposite effects on neural responses, where expectation reduces neural responses^32^. Such reduction is accounted for by the free energy principle^33^,^34^, according to which the brain continuously predicts stimulation, and thus increases in neural activity represent deviations from expectations.

For all these approaches, sensitivity to temporal irregularities should increase as a function of the number of stimuli composing a sequence^19^,^20^,^21^,^22^,^23^. Moreover, according to interval-based models, the presence of a stimulus sequence should not have an influence on perceptual judgments based on the *perceived* *timing* of individual stimuli; only tasks based on perceived duration should be affected. On the other hand, entrainment^13^ and predictive-coding models^33^,^34^, which are based on time-point representations, predict that expected stimuli could be perceived earlier than unexpected ones, a phenomenon called prior entry^35^. Here we reason that if a regular sequence leads to the expectation of a stimulus, then not only stimuli presented when expected but also stimuli presented later on should be expected and thus they should be perceptually accelerated^36^. On the contrary, several approaches have suggested that there should be no difference in the perceived timing of early and late stimuli^31^,^37^,^38^. We wanted to disambiguate such predictions and characterize the ways in which presenting stimuli in a sequence influences perceived timing. To do this, we asked participants to estimate the perceived timing of events embedded in a regular sequence either by reporting their regularity^22^ or by reporting their order with respect to a probe stimulus in another sensory modality. In comparing the results obtained with these two paradigms, we assume that the sequence could have an influence on the expected timing of stimuli thus influencing judgments of regularity. Instead, perceptual distortions due to the sequence should not have a marked influence on the perceptual latency of the probe stimulus if presented in another sensory modality^39^. As such, we assume that perceived timing is consistently measured using different tasks, but the reference to which timing is compared to could be differentially affected by the sequence (however see studies employing multiple tasks, where either response bias^40^ or the underlying representation of time is thought to be different^41^,^42^,^43^,^44^,^45^,^46^).

Behavioral and neurophysiological findings evidence that human and non-human primates reproduce temporal intervals in a way consistent with Bayesian inference^7^,^8^,^47^. It has been hypothesized that similar Bayesian accounts apply to perceived interval timing^48^,^49^,^50^, but empirical support is lacking. Such evidence is necessary, as timing information for motor and perceptual tasks is processed by separate systems^51^. In addition, models of perceived duration do not predict changes in perceived timing of individual stimuli following Bayesian inference as they are based on the representation of interval durations. Given recent calls to establish such accounts for perceived timing^48^,^50^,^52^, here we provide behavioral evidence that is consistent with the predictions of a dynamic Bayesian inferential process that operates for event-timing at a trial-by-trial level^53^ (see work in the sensorimotor synchronization literature for a similar event-based approach^54^,^55^,^56^,^57^). A perceptual estimate of individual stimulus timing is obtained at each point in time through the iterative combination of incoming sensory information and expectations of a stimulus based on previous intervals. A fundamental aspect of our model is that – differently from what happens at the interval level^7^,^8^,^49^ – probability distributions about timing are *asymmetric* due to the way time flows, and this asymmetry leads to a progressive perceptual acceleration of expected stimuli as the neural response becomes more tuned (i.e., with a shorter tail) for stimuli presented at the point of expectation.

## Results

### Behavioral results: Asymmetric temporal deviation detection

**Figure 1:**
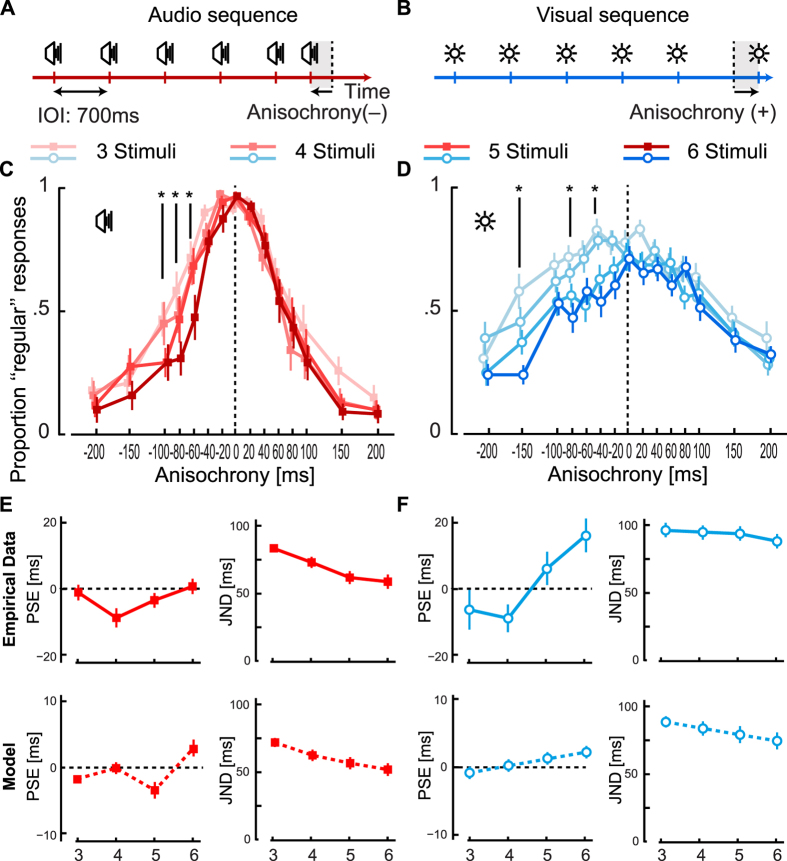
Stimuli and results of Experiment 1. Each participant is only presented with sequences of stimuli in one modality. (**A**) Example of an audio sequence with the final stimulus presented early. (**B**) Example of a visual sequence with the final stimulus late. (**C**) Proportion of “regular” responses as a function of the anisochrony of the last stimulus in an audio sequence and (**D**) in a visual sequence. Each line represents data obtained with a different sequence length. The distribution of responses is steeper with longer sequences (interaction term of a two-way repeated measure (r.m.) ANOVA on the inverse-normal proportion of “regular” responses bounded between 0.01 and 0.99; audio: *F*(42, 588) = 1.8, *p* = 0.0016, *η*_*p*_^2^ = 0.11; visual: *F*(42, 588) = 1.5, *p* = 0.0135, *η*_*p*_^2^ = 0.10). Asterisks denote anisochronies at which the proportions of responses significantly differ across the four sequence lengths (one-way r.m. ANOVA Bonferroni corrected, *p* < 0.0033). In all graphs, error bars represent the standard error of the mean. (**E,F**) Average point of subjective equality (PSE) and just noticeable difference (JND). The upper panels represent the values obtained from the empirical data using the Spearman-Kärber method^87^ (see Methods) showing that PSE values differ across sequence lengths both for auditory (one way r.m ANOVA; F(3, 42) = 6.0, p = 0.0017, *η_p_*^2^ = 0.30) and visual sequences (F(3, 42) = 6.4, p = 0.0011, *η_p_*^2^ = 0.31). The ability to discriminate ‘’regular” stimuli is higher for audio sequences supporting the idea that auditory stimuli have greater temporal resolution^58^. Such performance increases with longer sequences^19^,^21^,^22^,^92^, as exhibited by the negative course of JND values for auditory (*F*(3, 42) = 9.8, *p* = 0.0001, *η_p_*^2^ = 0.41) but not for visual sequences (*F*(3, 42) = 1.8, *p* = 0.1694, *η_p_* ^2^= 0.11)). The lower panels represent the predictions of the Bayesian asymmetric model.

In Experiment 1 participants judged whether the timing of the last stimulus in a sequence was regular or irregular. As one would expect, Fig. 1C,D shows that the proportion of sequences reported to be regular decreases with larger anisochronies, and such a pattern is more marked for audio sequences, reflecting the higher reliability of temporal judgments with auditory stimuli^58^. As described in the literature, sensitivity to temporal deviations increases with longer sequences^19^,^20^,^21^,^22^,^23^, but here we find that changes are present for stimuli presented earlier than expected: an asymmetry in anisochrony detection (Fig. 1C,D). A similar change in responses for early stimuli is also evident in an experiment performed with blocked presentation of sequence lengths rather than interleaved (Supplementary Fig. S1). From the response distributions we determined the anisochrony necessary for the perception of on-time judgments. We find that with short sequences, stimuli need to be presented a few milliseconds before the expected timing to be perceived as isochronous; with long sequences, the stimuli need to be presented later – up to 20 ms later than expected with sequences of six visual stimuli (Fig. 1E,F). In accord with these data, the effect persists when ‘early’ or ‘late’ judgments are used to assess changes in perceived timing^22^. As expected^19^,^20^,^21^,^22^,^23^, we also find that the width of the distribution (which is inversely related to the ability to discriminate if a stimulus is isochronous) decreases with longer sequence lengths (Fig. 1E,F).

### Behavioral results: Changes in perceived timing

**Figure 2:**
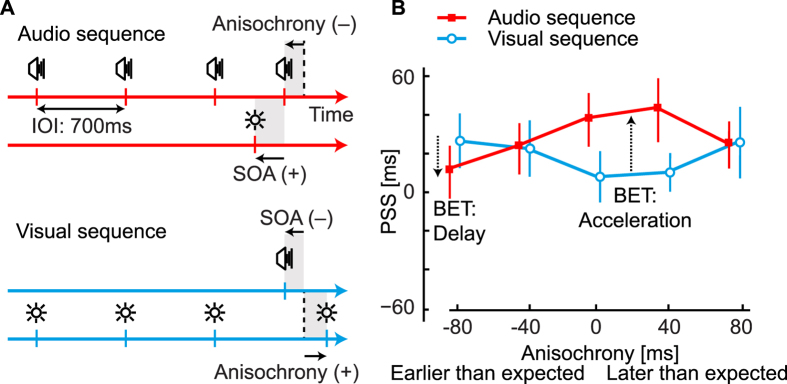
Stimuli and results of Experiment 2. (**A**) Examples of trial sequences where participants judged the temporal order of the audiovisual pair presented at the end of a sequence. Auditory and visual sequences were interleaved. Top: An audio sequence with the final stimulus presented earlier than expected (negative anisochrony) and with a light presented before the final audio stimulus (positive SOA). Bottom: A visual sequence with the final stimulus presented later than expected (positive anisochrony) and with sound presented before the final visual stimulus (negative SOA). (**B**) Average PSS values corresponding to the SOA at which audio and visual stimuli are perceived as being simultaneous. On the y-axis, a positive PSS means that light has to be presented before the sound to be perceived as simultaneous, whilst a negative value means that the sounds has to be presented before the light to be perceived as synchronous. The difference between PSS values on the two curves indicates the bias by expected timing: in this graph perceptual acceleration happens when the audio PSS is higher than the visual PSS. If there was no change in perceived timing across the presented anisochronies, the pattern of PSS values should be horizontal. The BET, instead, changes as a function of anisochrony (interaction term of a two-way r.m. ANOVA, *F*(4, 44) = 4.8, *p* = 0.0026, *η*_*p*_^2^ = 0.30) as stimuli presented at −80 ms are perceptually delayed whereas stimuli presented at 0 ms and +40 ms are perceptually accelerated.

In order to determine whether the results obtained in isochrony judgments are related to a change in the perceived order of stimuli, in Experiment 2 we employed a novel experimental paradigm where the last stimulus in a four-stimulus sequence is paired with a stimulus in another modality and participants reported the temporal order of this audiovisual pair (TOJ, Fig. 2A). For a review of the literature on temporal order judgments, please refer here^59^. From the response distributions we determined the audiovisual asynchrony necessary for the perception of subjective simultaneity (PSS). The last stimulus in the sequence could be presented on time or anisochronously (earlier or later than expected). To interpret the data, we make the assumption that the changes in PSS reflect changes in perceptual latency, although this is an unresolved issue^39^,^44^,^46^. In fact, if we assume that the sequence is more likely to exert an influence on stimuli of the same modality, then *changes* in PSS due to the anisochrony indicate a modification of the time at which the final stimulus is perceived (Fig. 2B); we call this effect *bias by expected timing* (BET). In particular, the comparison of PSS values obtained with audio and visual sequences evidences that if the last stimulus in the sequence is presented slightly earlier than expected, the BET leads to a later perception of the stimulus (delay). On the other hand, for the last stimulus presented at the expected point in time or later than expected, the BET leads to an earlier perception of the stimulus (acceleration). Physically synchronous audiovisual stimuli are differentially reported as either “sound first” or “light first” dependent on their anisochrony with the sequence as shown in Supplementary Fig. S2B. In addition, the BET effect is independent of the sequence modality (Supplementary Fig. S2A), and we find no difference in the discriminability between the audiovisual pair used in Experiment 2 (indexed by the just-noticeable difference (JND); see Methods) both across conditions or between modalities (Supplementary Fig. S2C).

### Behavioral results: Longer sequences and different IOIs

**Figure 3:**
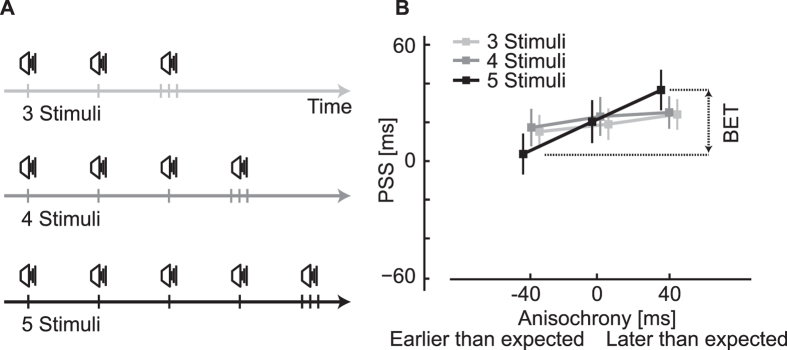
Stimuli and results of Experiment 3. (**A**) Examples of sequences of different lengths, where the last audio stimulus is paired with a visual stimulus (not shown). Differently from the results presented in Figs 2 and 3, here sequences are made only of sounds with a visual stimulus paired with the last stimulus in the sequence. (**B**) PSS values for early, on time, and late stimuli differ significantly, confirming the BET found in Experiment 2 (factor anisochrony of a two-way r.m. ANOVA, *F*(2, 46) = 7.9, *p* = 0.001, *η*_*p*_^2^ = 0.25). The magnitude of the BET increases with longer stimulus sequences (interaction of anisochrony and sequence length, *F*(4, 92) = 2.5, *p* = 0.049, *η*_*p*_^2^ = 0.10) and the effect is present with five stimuli (one-way r.m. ANOVA, *F*(2, 46) = 10.4, *p* < 0.001, *η*_*p*_^2^ = 0.31), but not with four and three stimuli (*F*(2, 46) = 1.6, *p* = 0.22; *F*(2, 46) = 0.60, *p* = 0.57).

To test whether the BET depends on the number of stimuli in the sequence, in Experiment 3 participants judged the temporal order of an audio and a visual stimulus following the presentation of audio sequences of different lengths (three, four, or five repeated stimuli presented in different blocks, Fig. 3A). Results indicate that the BET increases as a function of sequence length (Fig. 3B).

**Figure 4:**
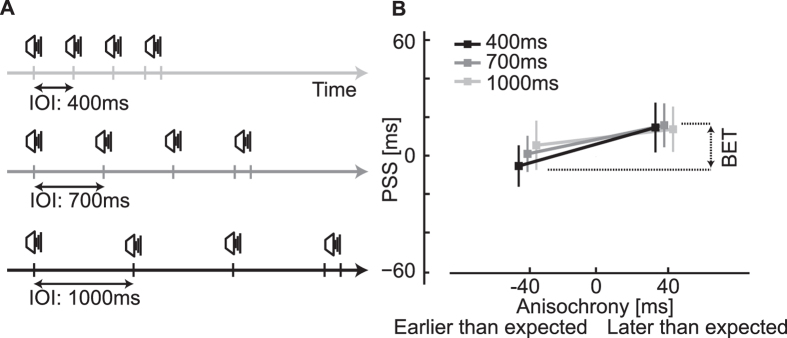
Stimuli and results of Experiment 4. (**A**) Examples of sequences with an inter-onset interval (IOI) of 400 ms, 700 ms, and 1000 ms where the last audio stimulus was anisochronous (±40 ms) and paired with a visual stimulus (not shown). Only auditory sequences have been presented. (**B**) PSS values indicate a BET similar to the other experiments (factor anisochrony of a two-way r.m. ANOVA, *F*(1, 23) = 15.7, *p* = 0.0006, η_p_^2^ = 0.41), which suggests that testing the same IOI throughout the experiment is not necessary to elicit the BET and that the effect is not limited to one IOI.

Furthermore, to test whether the observed effects are due to the repeated presentation of the same interval across all trials, in Experiment 4 we used trial sequences with four stimuli each but with varying inter-onset intervals (IOIs) interleaved within a block (Fig. 4A). The BET is still present when stimuli having different periodicities are interleaved in the same experiment (Fig. 4B).

### A Bayesian model of perceived timing

We model the results collected using *Bayesian decision theory* (*BDT*). Such a framework has been successfully applied to several perceptual domains^60^,^61^,^62^,^63^,^64^, including interval estimation^48^,^49^,^65^ and reproduction^7^,^8^,^14^, but here for the first time we propose a descriptive model that captures changes in the perceived timing of individual stimuli.

**Figure 5:**
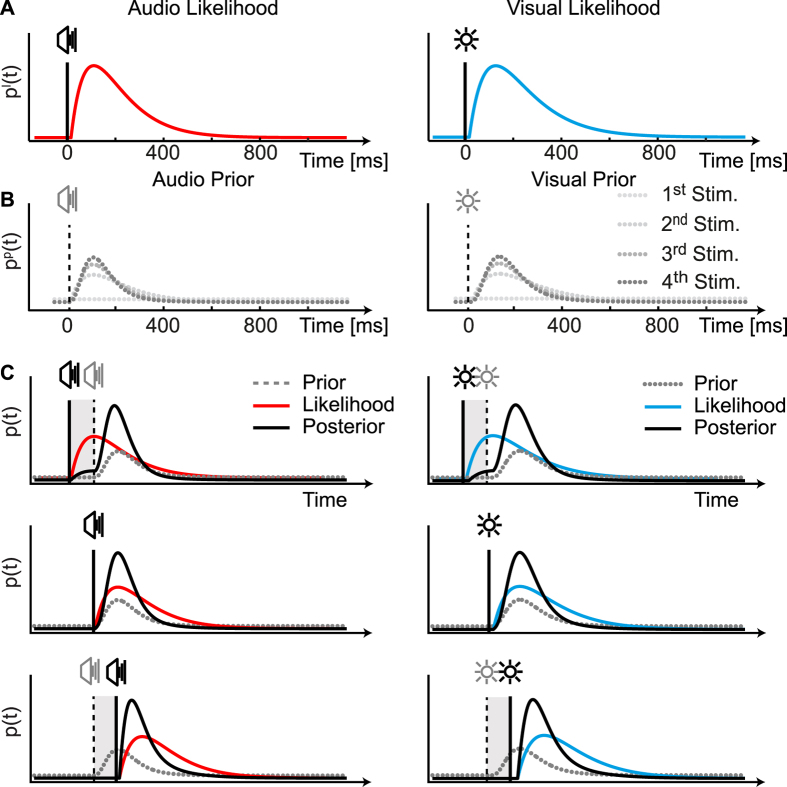
Bayesian model of perceived stimulus timing with asymmetric probability distributions (see Methods for details). (**A**) The top panel shows the likelihood probability distribution – the probability of sensing the stimulus presented at *t* = 0. The distribution is obtained by through an exponential low pass filtering of the input signal^66^. (**B**) The prior probability distribution for the next stimulus is obtained by combining the prior for the previous stimulus with the current posterior distribution, plus a constant (Methods, Equation 7). (**C**) Integration of prior and likelihood to obtain the posterior distribution according to Equation 6 for the fourth stimulus (last stimulus in Experiment 2) appearing −40 ms, 0 ms, or +40 ms with respect to the expected time (separate rows). Perceived timing is obtained retrospectively by considering the overall posterior distribution (i.e., by computing the mean of the distribution).

To do this, we hypothesize that the brain represents the probability of experiencing the onset of an event over time. To give an example, we can represent the timing of clapping sounds as the probability of *perceiving* a clap at any point in time (past, present, and future). The *sensing* of a clap happens necessarily after a delay, due to the filter characteristics of sensory channels^66^. The probability of sensing the clap increases at points in time immediately following the clap (likelihood probability, Fig. 5A). Due to the regular timing of applause, the probability of *encountering* another clap increases at regular intervals following the first clap (prior probability, Fig. 5B). If we extend BDT to the time domain, the likelihood (probability of sensing) and prior (probability of encountering) should be combined at each point in time (Equation 6) leading to the posterior probability (Fig. 5C). We will now examine the three components of this process: likelihood, prior, and posterior.

The *likelihood function* captures the probability of sensing a stimulus after it has occurred. As such, it represents temporal smearing due to delays in sensory processing, and therefore it is equivalent to the impulse response function^67^. In other applications of BDT to temporal properties, the likelihood has been assumed to have a Gaussian distribution over time^7^,^8^,^14^,^48^,^49^,^65^, but here we propose that the likelihood should have an asymmetric shape because of the intrinsic constraints of sensing individual stimuli over time. First, time flows in one direction, and thus the causality of sensory processing needs to be directional. As such, the probability of a stimulus being sensed is more than 0 only after a delay due to neural processing. Thereafter, because sensory processing can only last a finite amount of time, the likelihood probability should return to 0 (unless the stimulus could be missed). We propose that the probability of sensing a stimulus at time *t* can be captured by a monophasic impulse response function resulting from an exponential low-pass filter^66^ (Equation 8). Figure 5A shows the shape of the distributions that capture the results obtained with an audiovisual temporal order judgment task. If we assume that exactly one perceived onset is associated with a stimulus, then the probability of perceiving the stimulus at any point in time should sum to 1, and in this way we can deal with two *likelihood probability distributions* (instead of likelihood functions that are commonly used in Bayesian models).

Here we further assume that the impulse response function remains unchanged with successive stimuli whilst the shape of the *prior probability distribution* changes. The *a priori* probability of a stimulus over time is modeled to be flat when the first stimulus is presented (Fig. 5B). After the first stimulus occurs, the prior should not be flat anymore due to knowledge of the temporal statistics of the environment. To understand why, again consider hands clapping. When do you expect the second clap to occur? The probability of the *second* stimulus occurring *before* the first one is necessarily nil: the prior starts at 0 when the first clap is heard, and it increases in the future. The most probable time at which you expect a second clap corresponds to the most frequently experienced interval between claps (roughly one fourth of a second^68^). The probability of hearing a second clap then decreases over time but does not reach 0, as hearing a clap tomorrow is always a possibility. Here we assume that the prior for the second stimulus peaks at the most frequent inter-onset interval used in the experiment (700 ms).

When the hands clap for the second time, the perceptual system has an estimate of the duration of the interval between two successive claps. From previous experience there is the knowledge that subsequent intervals are likely to be similar in duration (clapping variability is typically 2.5% of the intervals^68^). Because of the small variability in timing of clapping and of other similar isochronous sequences, here we assume that successive intervals within a single sequence are expected to have the same IOI. The relationship between successive intervals has been instead modeled probabilistically (as the likelihood probability distribution) in Bayesian models of interval estimation^7^,^8^,^49^.

Temporal expectations build up as more information is acquired. To model this, we update the prior probability in a way similar to a Kalman filter, by recursively integrating the posterior distribution of the previous stimulus into the prior (Equation 7). The prior distribution becomes more and more similar to the asymmetric likelihood, while its maximum value does not deviate from the previously experienced intervals (Fig. 5B). As the posterior is produced by the asymmetric prior and likelihood, its right side is also longer than the left, but this asymmetry decreases at every stimulus. Rather than considering the maximum posterior distribution as reflecting perceived timing, here we propose that the whole shape of the posterior probability distribution over time is considered (see also a recent paper that uses a similar way of calculating sensory estimates^69^). The pattern of BETs is due to the combination of the asymmetric likelihood with the asymmetric prior: there is an attraction of the posterior towards the prior, but the larger reduction of the posterior’s right tail can account for the perceptual acceleration of expected stimuli compared to the likelihood taken alone (Fig. 5C, middle).

**Figure 6:**
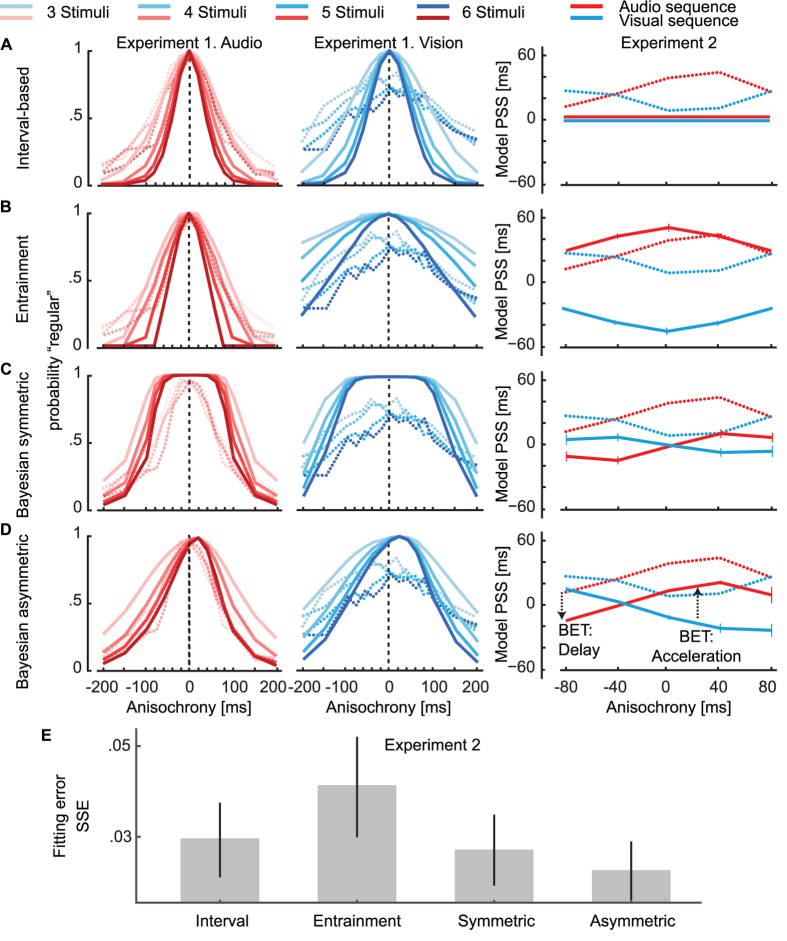
Comparison of the model predictions for Experiments 1 and 2 (see Methods for details about their implementations). The data from Experiment 1 & 2 is overlaid to the predictions of each model. (**A**) Predictions of an interval-based model obtained by fitting the multiple-look model^20^ to the distribution of responses. The model does not predict asymmetries in Experiment 1 or changes in perceived timing in Experiment 2 as it is based on representation of durations. (**B**) Prediction of an entrainment model^22^. Detection of irregularities has been hypothesized to be symmetrical^7^,^13^,^36^,^37^, and the predictions for Experiment 1 suggest only a slight asymmetry (that is more evident in the visual condition). The model does not make explicit predictions about changes in perceived timing of stimuli, but here we show how a 15 ms prior-entry effect^34^ proportional to the attentional pulse would affect the results of Experiment 2. (**C**) Predictions of a Bayesian model with Gaussian likelihood distributions. The posterior is obtained by combining likelihood and prior while the likelihood of the last stimulus is shifted according to the presentation anisochrony. The predictions for a distortion in perceived timing of stimuli obtained in Experiment 2 are used to modify the conditions of the interval-based model in Experiment 1. It should be noted that there is no BET for isochronous stimuli due to the symmetry of the distributions. (**D**) Prediction of a Bayesian model with asymmetric distributions (Fig. 5). PSS for Experiment 2 are obtained from the posterior of the audio and visual stimuli (see Fig. 7). The pattern indicates that there is no BET for stimuli presented roughly 40 ms earlier than expected. Predictions for Experiment 1 are obtained by modifying the timing of stimuli in the interval-based model. (**E**) Goodness of fit measures for each model. We minimized the sum of the squared error (SSE) between the empirical data and the output from each model. We find that the Bayesian model with asymmetric distributions best captures the data.

Figure 6 shows the results of a simulation for the Bayesian model we propose, as well as the interval-based and entrainment models we have discussed in the introduction (see Methods for details about their implementation). The predictions of each model for the conditions of Experiments 1 should be compared to the experimental data summarized in Fig. 1. The data of Experiment 1 and 2 is overlaid to the results of each model in Fig. 6.

## Discussion

Our psychophysical experiments show that temporal regularity can change the perceived timing of stimuli – the *bias by expected timing* effect (BET) – without requiring participants to perform speeded responses (that can be affected by motor preparation) nor magnitude estimation (that can be subject to behavioral optimization^52^). The results of Experiment 1 are obtained by asking participants to judge the regularity of a stimulus with regard to the sequence’s IOI. These data show that, with longer sequences, stimuli need to be presented later than expected in order to be perceived as isochronous. The pattern of results across sequence lengths is consistent with findings where participants were asked to choose whether the final stimulus was presented early or late compared to expectation^22^. In both paradigms, participants could perform the task by comparing the perceived timing of stimuli to the expected timing (or by comparing the perceived duration of the last interval with a stored average of the intervals in the sequence^19^ but if this was the case we should not have found a consistent bias). We should consider that if the task is based on the expected timing of future stimuli, then such a prediction should be based on the perceived timing of previous stimuli. Thus, if the perceived time of stimuli is distorted, then also the expected timing cannot be veridical. In other words, we have reason to believe that the asynchrony required for maximum perceived isochrony should be less than the actual BET, because perceived regularity results from a combination of distortions in perceived timing and distortions in expected timing. On the other hand, in Experiment 2 participants were not required to compare the last stimulus to expectations, but to a stimulus in another modality, which is presumed to be unaffected by distortions occurred in the sequence stimuli. Such a paradigm thus gave us a less biased measure of the BET. In addition, it allowed us to register changes in perceived timing as a function of anisochrony. The results of Experiment 2 indicate that the BET leads to an acceleration of stimuli presented at the expected time point or later. In addition, the BET for stimuli presented earlier than expected induces a perceptual delay. The magnitudes of the effects found in Experiment 2 are larger than the ones found in Experiment 1. It has been reported that longer sequences lead to better discrimination of anisochrony^19^,^20^,^21^,^22^,^23^, and accounts of temporal sensitivity present in the literature predict symmetric performance for early and late stimuli^23^,^31^,^37^,^38^. However, there have been no studies that have tested this prediction, although “slight asymmetries” in the profile of data have been previously described^37^. Our results clearly show asymmetric performance.

An open issue is whether the BET found in the experiments is due to acceleration and slowing down of perception, or, alternatively, if a post-dictive inference process at the decisional level can account for the results^70^. Given that evidence exists to show faster processing of attended stimuli^26^,^35^,^37^, as well as evidence for post-dictive inference of sensory properties^70^, the truth may lay somewhere in between. Thus, the disambiguation between generalized perceptual acceleration and decision-based inference processes is thus of primary concern for future empirical work.

The BET counteracts the improved detectability of stimuli presented later than expected; that is, stimuli following a long sequence that are presented later than expected are perceptually accelerated (leading to an increase of “regular” responses) against the detectability of the asynchrony (which should lead to an increase of “irregular” responses). Stimuli presented isochronously are instead perceptually accelerated both in cases where participants reported if stimuli are perceived to be isochronous (Experiment 1) or whether the final stimulus appears to be presented early or late^22^. The magnitude of the acceleration of isochronous stimuli is very similar when measured with these two tasks, but it is somewhat smaller than the acceleration effect found with the temporal-order judgment paradigm of Experiment 2. On the other hand, from the results of Experiment 2, we see that perceptual delay is only present at large anisochronies for stimuli presented earlier than expected (larger than the point where the two curves cross in Experiment 2 – around 40 ms as in Fig. 2B). Thus the BET for early stimuli is insufficient to counteract the effect of the improved detectability, leading to an asymmetric distribution of responses. Figure 6 allows us to quantitatively compare the predictions of extant models of time perception to our proposed model. We find that the Bayesian model with asymmetric probability distributions provides the best fit to the data when comparing the goodness of fit for each model (Fig. 6E).

*Interval-based* models explain perceptual effects related to the presence of rhythmic sequences through a modification of the representation of the interval duration^21^,^71^. While the model accounts for an increase in the sensitivity to temporal deviations (Fig. 6A), such a predicted increase is necessarily symmetrical and thus cannot account for the experimental data. Furthermore, the model does not predict changes in the perceived timing of stimuli at different anisochronies as it is based on the representation of unbiased interval durations.

To quantify the predictions of *entrainment models*, we simulated an eminent model tailored to the experimental paradigm employed in Experiment 1^23^. We find that the detection of irregularity does not follow the asymmetric pattern of Experiment 1 (Fig. 6B). Entrainment models could be formulated to predict changes in perceived timing of stimuli by appealing to the prior-entry effect^35^ as a function of temporal attention^23^,^37^,^72^. The outcome is a symmetric acceleration that decreases with deviant stimuli (Fig. 6B). It should be noted that in the original formulation, the detection of irregularity has been thought to be unaffected by this temporal distortion^23^.

The *Bayesian* model with *symmetric* distributions predicts that the perceived timing of irregular stimuli should be biased to make any deviant intervals more similar to previously experienced ones^48^,^49^. The magnitude of the bias decreases with large anisochronies, and the effect is identical for stimuli presented too early and too late, leading to a symmetric pattern in Experiment 2 (Fig. 6C). The distortion in perceived timing towards isochrony should make the detection of anisochronies more difficult, leading to a wider (and symmetric) distribution of responses in Experiment 1.

The *Bayesian* model with *asymmetric* distributions is based on the relaxation of the normality assumption often employed in BDT accounts^7^,^8^,^14^,^48^,^49^,^73^. As for the prior-entry phenomena^74^, perceptual acceleration for on-time and late stimuli and perceptual delay for early stimuli are explained through changes in the shape of the posterior – not by a shift of the distribution^44^. In this way, the absence of a BET is predicted for stimuli presented earlier than expected, not for isochronous stimuli. The asymmetry in the BET makes the predicted pattern of perceived timing of stimuli, shown in Fig. 6D, qualitatively match the pattern of results visible in Fig. 2B. The model predicts a temporal regularization, as in recently proposed models of interval estimation and reproduction^7^,^8^,^14^,^48^,^49^,^73^, as interval duration estimates are computationally successive to the estimate of individual stimulus timings^75^. We propose that such regularization could be seen as a modulation of the prior-entry effect as a function of the survival probability^36^,^76^. The distortion in perceived timing also generates better discrimination of temporal irregularities for earlier than for late stimuli in long sequences, which resembles the pattern found in Experiment 1 (Fig. 1B; Supplementary Fig. S1). In sum, the asymmetric Bayesian model accounts for the data of the two experiments.

The asymmetric model, perhaps counterintuitively, predicts that the BET should not vary substantially if the sequence is composed of different stimuli (i.e., sounds vs. lights) as we assume similar processing mechanisms across modalities^64^. Because the prior resembles the likelihood, and the BET is due to the ratio between the width of the prior and the width of likelihood, the ratio between the two widths remains roughly constant. For this reason, BET curves have similar patterns for different stimulus types. The difference between stimuli becomes evident as a modulation in the tuning of the effect (i.e., the spread of the BET across anisochronies). As we find no effect of anisochrony on PSS with four stimuli in Experiment 3, but the same condition leads to a BET effect in Experiment 2, we hypothesize that the difference can be due to an *a priori* probability distribution with heavier tails (i.e., a higher value of added constant *ω*, see Methods), which would be justified as knowing the type of stimuli would decrease attentional demands (see^22^ for a similar explanation regarding unpredictable stimuli). The pattern of results found in Experiment 1 may instead be explained by the combination of the diminishing asymmetry and increasing precision of the posterior distribution. This means that the asymmetry in the data should be most evident for a sequence composed of a limited number of stimuli. The predictions of the asymmetric model for Experiment 1 (made with parameters fitted to the data of Experiment 2) capture qualitatively the pattern of results, but the magnitude of the change in PSE is smaller than the data (Fig. 1E,F). This difference could be also explained by the shape of the prior (which is modulated by the added constant *ω*). Because of the shape of the prior over time, the model naturally accounts for perceptual phenomena related to the scalar property of interval timing (the estimation error of an interval increasing as the IOI increases^17^,^18^,^71^), and with longer intervals the prior becomes flatter leading to a smaller BET (Fig. 4).

Although the proposed Bayesian model requires the full specification of the probability distributions over time before a perceptual decision is made, the formulation could be extended to account for just-in-time responses, i.e. responses given before the probability distributions associated with each stimulus have completely unfolded. In such a case, perceptual decisions could be performed using only the probability distributions specified until the current moment in time, but such responses would deviate from optimality. Our data, however, shows that if these responses existed, they are rare in the experiments reported here, as response times measured from the first of two stimuli (1238 ± 94 ms in Experiment 2) are, on average, longer than the combined maximum level of SOA (350 ms) plus the time required for the full probability distribution (around 500 ms). Moreover, it has previously been reported that participants take more time to answer difficult tasks in temporal perception^77^, thus too-fast responses should not be showing up in cases where they could actually influence performance, i.e. at anisochronies near the threshold in Experiment 1 and at SOAs near PSS in Experiment 2.

Stimuli in our experimental paradigm conform with the natural statistical tendency of successive intervals to be similar in duration^78^. We hypothesize that the effect of temporal regularity on perceived timing can be described as the influence of a prior having a shape that is quickly updated within a regular sequence of stimuli. Perceptual effects thus become readily evident without the need to present the same property throughout the experiment^7^,^8^. The effect of such rapid updates of the prior are in line with the findings of bottom-up influence of regular sequences on perception^72^ and with changes in simultaneity judgments after exposure to only one audiovisual stimulus^53^. Changes in simultaneity perception have also been related to changes in perceived timing of individual stimuli^44^, which in some accounts have been explained by changes of the likelihood function^79^ rather than by the influence of an asymmetric prior as proposed here.

Several accounts of temporal perception hypothesize that incoming sensory information is compared to a memory component, where the average interval between stimuli is stored^18^,^19^. Bayesian models of perceived duration have suggested that such a component captures the *a priori* probability distribution^49^,^80^. Similarly, the Bayesian model we propose requires the representation of the a-priori probability of perceived timing. The nature of the task suggests that the dynamic formation of the *a priori* probability distribution could be implemented neurally by the iterative entrainment of cortical activity, leading to tuned attentional deployment at an expected time point^9^,^13^,^31^. As such, the phase of delta-theta activity could be a plausible neurophysiological correlate of for representing the *a priori* probability of encountering a stimulus^81^, and recent work supports the idea that facilitation of sensory processing is shaped by priors^11^. Further, temporal expectations have been shown to lead to a desynchronization of alpha-band activity^9^, where the neural response to stimuli is amplified at the expected time point leading to modulations of perceived timing^74^. The proposed model is in line with this finding, as stimuli that are presented too early are not amplified because they come in before amplification has been activated. Stimuli that are presented on time or too late are instead amplified leading to a perceptual acceleration. We should consider, however, that secondary neural populations may also be active with stimuli presented later than expected, registering the violation of expectations^82^,^83^. Separate from expectation, the activity of the secondary populations should increase over time^36^,^76^,^84^. The interplay between the two types of responses could result in the dual effect of regularization and anticipation on perceived timing^85^. The Bayesian model we present accounts for the conjoint effect of expectation-based activity^86^ and violation-based activity by relying on the asymmetry of the prior distribution.

## Methods

### Ethics statement

The STEM Ethics Committee of the University of Birmingham approved the study and all experimental protocols. The methods were carried out in accordance with approved guidelines.

### Participants

In total 90 undergraduate students participated in the study with an average age of 20.83 (SD: 2.20). For Experiment 1, 15 students participated in the auditory experiment (10 females, M_age_ = 21.07, SD_age_ = 1.87) and 15 in the visual experiment (9 females, M_age_ = 20.27, SD_age_ = 1.83); Experiment 2 involved 12 participants (10 females, M_age_ = 20.67, SD_age_ = 2.50); Experiment 3 involved 24 participants (18 females, M_age_ = 21.17, SD_age_ = 2.53); and Experiment 4 involved 24 participants (16 females, M_age_ = 20.67, SD_age_ = 2.16). All participants gave informed consent prior to the experiment, and they were either compensated £6 per hour or given course credits. All reported normal or corrected-to-normal hearing and vision, and they were all naïve to the purpose of the experiment.

### Experimental setup

Participants sat in a quiet, well-lit room at a distance approximately 50 cm from the light- and sound-producing apparatus. A red 5 mm LED positioned in front of the participant (20 ms with 5 ms linear ramp, 91 Cd/m^2^) produced visual stimuli. A speaker 50 cm to the left of the participant (20 ms with 5 ms linear ramp, 1 kHz, 75.1 dBA) produced audio stimuli. A computer audio card connected to two identical audio amplifiers generated signals, all of which were loaded onto the audio card before the trial started to ensure accurate timing.

### Psychophysical procedures

#### Experiment 1 – Isochrony judgments

The aim of Experiment 1 was to test whether there is an increase in sensitivity to temporal deviations as a function of how many stimuli there are in a sequence. Fifteen participants took part in the audio experiment and another 15 in the visual experiment. Sequences of three, four, five, or six unimodal stimuli (either audio or visual) were presented with a regular inter-onset interval (IOI) of 700 ms, except the last stimulus, which had a deviation of 0, ±20, ±40, ±60, ±80, ±100, ±150, or ±200 ms. Each trial type was repeated eight times. The participant’s task was to report whether the last stimulus appeared to be regular or not with the rest of the isochronous sequence. Participants responded by pressing one of two keys, and the next stimulus would appear 1.5 to 2 s after the keys had been released. For each participant, we computed the proportion of responses for each anisochrony and sequence length. Individual trials for different conditions were randomly interleaved in all experiments.

#### Experiment 2 – Audiovisual temporal order judgments

The goal of Experiment 2 was to understand whether the anisochrony at which a stimulus is presented affected the perceived timing of a stimulus in a sequence. Participants completed the experiment in two phases: the *practice phase* and *test phase*. The goal of the practice phase was to familiarize participants with the audiovisual temporal order judgment (TOJ) task, assess performance, and provide baseline data for the creation of the Bayesian models. Participants were presented with a single audiovisual stimulus pair separated by a stimulus-onset asynchrony (SOA) of 0, ±20, ±90, ±170, ±250, or ±350 ms. Each SOA was repeated six times, totaling 66 trials. The participant’s task was to report whether the audio or visual stimulus appeared first in time. Participants responded by pressing one of two keys, and the next stimulus would appear 1.5 to 2 s after they had been released.

During the test phase, participants were presented with a unimodal (either audio or visual) sequence of four stimuli having an IOI of 700 ms, except the last stimulus, which deviated by either 0, ±40, ±80 ms. The last stimulus in the sequence was presented together with a stimulus in the other modality (e.g., a visual stimulus paired with a sequence of sound stimuli) with an SOA of 0, ±40, ±80, ±120, or ±200 ms with respect to the anisochrony of the last stimulus presented. Each trial type was repeated eight times. The participant’s task was to report which of the two stimuli presented at the fourth point in time appeared first, i.e., audio first or visual first. Participants responded by pressing one of two keys, and the next stimulus would appear 1.5 to 2 s after they had been released (a review on TOJs is provided here^59^).

For each participant, we computed the proportion of responses for each presented SOA. Of particular interest to our hypotheses was the point of subjective simultaneity (PSS): the SOA at which an individual participant was equally likely to respond that either of the two stimuli was first. Positive PSS values mean that the light had to be presented before the sound to be perceived as synchronous, and negative values indicate that the sound had to be presented before the light for perceived synchrony. Changes in PSS as a function of anisochrony indicate a modification of the perceived timing of stimuli due to expectation. Also of interest was the just-noticeable difference (JND), the asynchrony necessary so that participants report the correct order of the stimuli at a proportion of .84 (which corresponds to 2σ). The PSS and JND were estimated as the first and second moments of the distribution underlying the psychometric function by using the Spearman-Kärber method^87^. This method provides non-parametric estimates that avoid assumptions about the distributions underlying the psychometric functions. A mathematical derivation of the method follows. First we define *SOA*_*i*_with *i* = {1, … 15} as the 15 values of audiovisual SOA used in the experiments and *p*_*i*_ with *i* = {1, *…* 15} as the associated proportion of “light first” responses. We further set two SOAs outside of the range tested, *SOA*_*0*_ = −250 ms*, SOA*_*16*_ = + 250 ms, to be able to compute the intermediate SOA between two successive ones


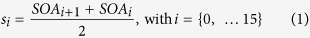


We then define two associated proportions to these extreme SOAs *p*_*0*_ = 0 and *p*_*16*_ = 1, and we calculated the associated values of the difference in proportion


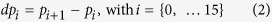


With these indexes we can express PSS and JND analytically as such:


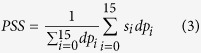


and


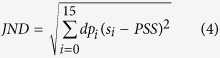


We used values of PSS and JND in the test phase of the experiment to assess participant performance. If JND was below 200 ms and if PSS did not exceed ± 175 ms, participants performed one of the experiments below. We used test-phase data to determine the likelihood distribution parameters of both the symmetric and asymmetric Bayesian models (detailed below) so this simple TOJ task was not biased by temporal expectations and thus reflected likelihood probabilities alone.

#### Experiment 3 – Number of stimuli in a sequence

Experiment 3 was aimed at measuring whether the changes in PSS found in Experiment 2 increase as a function of the number of stimuli in a sequence. Only one sequence length was presented in each of four blocks (the order was counterbalanced across participants). Sequences of three, four, or five audio stimuli were presented with an IOI of 700 ms, except the last stimulus, which had a deviation of 0 ms or ±40 ms. The last stimulus was presented together with a visual stimulus with an SOA of 0, ±40, ±80, ±120, or ±200 ms. Each trial type was presented 12 times.

#### Experiment 4 – Sequences with different periods

The goal of Experiment 4 was to check whether changes in PSS still occur if sequences don’t have the exact same period. Four types of audio sequences were presented with an IOI of 400, 700, or 1000 ms, except the last stimulus, which had a deviation of ±40 ms. The last stimulus was presented together with a visual stimulus with an SOA of 0, ±40, ±80, ±120, or ±200 ms. Each trial type was presented 12 times.

### Model fit and predictions

#### Interval-based model

It has been suggested that the precision of a duration estimate improves when multiple estimates are obtained from a sequence of stimuli. The perceptual system is hypothesized to be capable of averaging duration estimates in a statistically optimal fashion^19^. The multiple look model expands this analysis by quantifying the discrimination performance with two sequences of isochronous intervals and allowing for the differential contribution of the two sequences to the judgment^20^,^21^. We adapted the formula of the multiple look model to the conditions of Experiment 1 (for a derivation see^22^) so that we could estimate the JND obtained with intervals of *N* = {3, 4, 5} (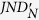) from the individual subject’s value of JND with the sequence of two intervals (*JND*_2_) according to:


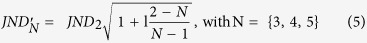


The weight parameter *l* was tuned by minimizing the sum of the squared differences between the observed data in Experiment 1 and the model for the audio and visual modalities. As such, the *l* parameter was 0.964 for audio and 0.958 vision. Predicted 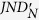 were used as parameters of Gaussian distributions of the responses (the maximum point of the curves was normalized to 1 for better comparison across the models). The mean response distributions across participants for each sequence length are shown in Fig. 6A. We then calculate JND by substituting the proportion of “regular” responses to the term *dp in* Equation 4. Interval-based models predict no changes in perceived timing of stimuli, leading to constant PSS values as a function of anisochrony. To quantitatively compare such predictions to our data, we found the sum of the squared error between a PSS of 0 for all conditions and the empirical data (Fig. 6A).

#### Entrainment model

We implemented the entrainment model for perceived temporal regularities^23^ and simulated 1000 sequences for each of the temporal deviations and sequence lengths used in Experiment 1. The probability distribution that simulates the results of Experiment 1 is shown in Fig. 6B (maximum point normalized to 1).

Entrainment models do not make explicit predictions about changes in the perceived timing of stimuli, but only on the amount of attention devoted at each point in time. To relate entrained attention to perceptual acceleration, we hypothesized a prior-entry effect^35^ that is proportional to the magnitude of the attentional pulse at the time the stimulus is presented^23^,^26^,^37^. We fitted individual parameters of the entrainment model by minimizing the sum of the squared error between the observed data from Experiment 2 and the model output to audio and visual sequences. This yields best fitting parameters^23^: period coupling *q* = 0.524, oscillation coupling *η* = 0.451, and the focusing parameter *κ* = 0.534. We also fit the magnitude of the prior-entry effect to the data, obtaining a value of 12.3 ms.

#### Bayesian symmetric model

Perception is obtained from the posterior distribution, i.e., the integration of the on-line sensory evidence (likelihood) with *a priori* knowledge of when a stimulus is expected to be sensed (prior). We propose that expectations are not static, but they are obtained by iteratively updating the probability of encountering a stimulus at each point in the future.

**Figure 7:**
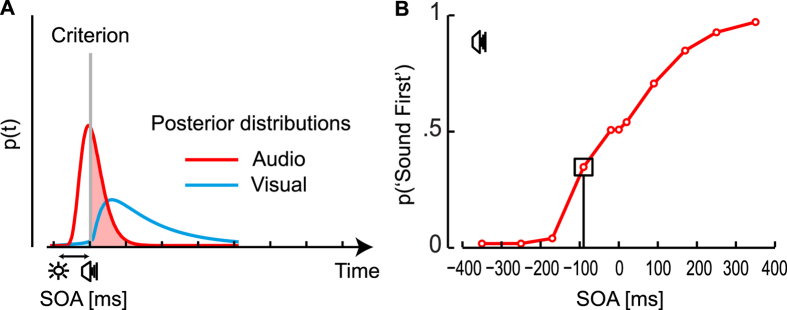
Example of how Signal Detection Theory is used to compute model responses across trials. (**A**) Across trials, the posterior distributions of the audio and visual stimuli can be considered to directly compute the proportion of sound-first responses. To translate the single trial decision rule across trials, one needs to search for the unbiased response criterion that gives the highest d’ between the two curves. For each asynchrony, the probability of “sound first” responses, after having identified such optimal decision rule across trials, is calculated as the sum of the two areas below the visual posterior on the left of the criterion (Hits) and below the audio posterior on the right of the criterion (CR). (**B**) The probability values of “sound first” responses obtained from the model for different SOAs are analyzed using the Spearman-Kärber method for participants’ responses (see Methods).

The likelihood probability distribution *p*^*l*^ (*t*) is the probability of sensing a stimulus at time *t* given that the stimulus is produced in the environment. Gaussian distributions with 0 mean and variance σ^2^ are used to describe the noise in sensory latency for each modality. We determined the value of the parameters *σ*_*A*_ and *σ*_*V*_ (subscripts *A* and *V* denote audio and vision, respectively) that give most similar values of obtained PSS and JND, as described in Fig. 7. We obtained the posterior probability distributions *p*^*q*^ (*t*) by multiplying the probabilities of the likelihood *p*^*l*^ (*t*) and the prior *p*^*p*^ (*t*)


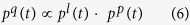


We obtained the prior probability distribution *p*^*p*^ (*t*) by using the posterior probability *p*^*q*^ (*t*) for the previous stimulus (i.e., *p*^*q*^ (*t*) for the time *t-IOI*). The added constant *ω* leads to a prior with heavy tails^88^ that allows sudden changes in IOI, and then decreases the tendency of fully incorporating the posterior into a new prior (thus mitigating the increase in false alarms^11^). This is expressed by:


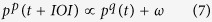


**Figure 8:**
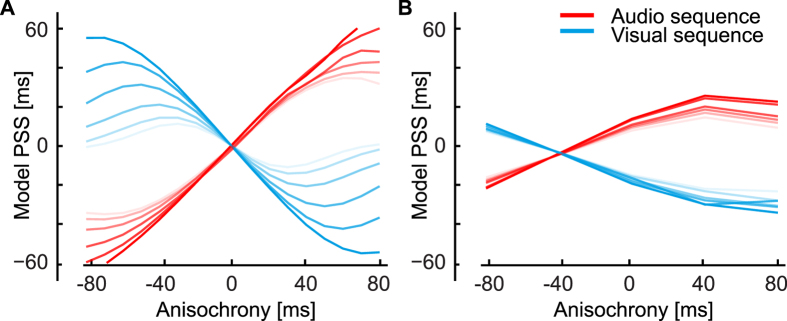
Predictions of the Bayesian models for Experiment 2 with different values of the added constant *ω*. Predictions obtained with lower values of *ω* are plotted with more saturated colors (*ω* = 0.032, 0.016, 0.008, 0.004, 0.002, 0.001). Higher values lead to flatter curves as the prior has less and less effect and the BET is smaller. (**A**) Bayesian model of perceived timing with symmetrical distributions and (**B**) with asymmetrical distributions.

The parameter *ω* changes the predictions of the model as shown in Fig. 8A,B.

To obtain the predictions for Experiment 2 we calculated the values of the posterior probability distributions for the last stimulus in the sequence, applying Equations 6 and 7 iteratively. Following previous empirical work^89^, we assumed that the brain does not only consider the onset of the stimulus to perform a TOJ. Although it is unclear what feature is considered for TOJs^39^,^90^, for computational simplicity we adopted the mean of the distribution (which is also in concert with recent work^69^). At each trial, the response is determined by the sign of the difference in timing between the means of the distributions to be compared^39^. A similar but computationally more tractable rule would be to calculate the difference in timing corresponding to an accumulated probability of 0.5 (i.e., the time corresponding to the median of the probability distribution). To calculate the proportion of responses across trials, we applied signal detection theory to the audio and visual posterior distributions over time^91^ (Fig. 7A). Several models of TOJ assume that differences in perceived relative timing are coded in the brain as the combination of presented asynchrony and latency difference in two channels^39^. The subsequent decision criterion is applied to this represented quantity. Here, we expand this approach by considering not only the representation of a single asynchrony value but of the whole probability distribution of asynchronies. The criterion then applies to a probability distribution and as such the decision is probabilistic leading to the proportion of responses as shown in Fig. 7B. From the proportion obtained at different asynchronies between audio and visual stimuli, we calculated the PSS using Equation 3. The value of the parameter *ω* influences the posterior and thus these proportions, and then subsequently modulates the amount of regularization as shown in Fig. 8A,B. We determined the value of *ω, σ*_*A*_ and *σ*_*V*_ that best fit the PSS results of Experiment 2 shown in Fig. 2B. We obtained *ω* = 0.0038, *σ*_*A*_ = 0.0142 and *σ*_*V*_ = *0.0405*. The best fit to the data is shown in Fig. 6C.

To derive the predictions for Experiment 1, we used the JNDs calculated from the interval-based model (Equation 5), to determine the standard deviations of the Gaussian curves of each sequence length. Before calculating the response probability distributions, we derived the temporal distortions for each anisochrony (horizontal-axis; Fig. 6A; left panels) given the Bayesian symmetric model generated in response to Experiment 2 (Fig. 6C; right). Thus, instead of representing the actual anisochronies, they represent the sensed stimulus timing.

#### Bayesian-asymmetric model

The likelihood probability distribution *p*^*l*^ (*t*) is modeled as a monophasic impulse response function due to an exponential low-pass filter^66^ expressed by


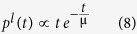


The proportional sign is due to the normalization across the whole distribution, which makes the area under the curve equal to 1. The prior probability distribution and posterior probability distribution are obtained as described for the symmetric model (Equations 6 and 7). The predictions for the asymmetric Bayesian model are presented in Fig. 6D, where the parameter *ω* modulates the BET as shown in Fig. 8A,B. We fit the *μ* parameter for audio and visual stimuli and the added constant 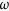 of Eq. 7 to the results of experiment 2: obtaining *μ*_*A*_ = 75.0 ms, *μ*_*V*_ = 87.0 ms and *ω* = 0.0009 (see Fig. 5A). The response distributions for Experiment 1 (Fig. 6D; left panels) were calculated in the same way as the symmetric model, however the temporal distortions applied were generated from the asymmetric model.

### Model comparison

For each model the parameter values were determined by minimizing the sum of the squared error (SSE) between the observed data and the model for both audio and visual modalities – for each participant. The SSEs for each model are presented in Fig. 6E. We found that the model with the best fit to the data was the Bayesian asymmetric model. We submitted the SSE values for each model to a one-way repeated measures ANOVA (corrected due to sphericity violation with the Greenhouse-Geisser correction) that was statistically significant *F*(1.097, 12.071) = 4.5, *p* = 0.05, *η_p_*² = 0.29). Similarly, a Bayesian repeated measures ANOVA showed strong evidence for a difference between conditions *BF*_10_ = 5.4. Post-hoc analysis showed that the Bayesian asymmetric model’s SSEs were significantly lower than the Bayesian symmetric model *Z* = 7.0, *p* = 0.009, *BF10* = 5.6, interval model *Z* = 0.0, *p* <0.001, *BF10* = 13.38, and entrainment models *Z* = 6.0, *p* = 0.007, *BF*_10_ = 2.5.

## Additional Information

**How to cite this article**: Di Luca, M. and Rhodes, D. Optimal Perceived Timing: Integrating Sensory Information with Dynamically Updated Expectations. *Sci. Rep.* **6**, 28563; doi: 10.1038/srep28563 (2016).

## Supplementary Material

Supplementary Information
