# Supplementary material for: Optimal Perceived Timing: Integrating Sensory Information with Dynamically Updated Expectations
Source: Sci Rep. 2016 Jul 7;6:28563. doi: 10.1038/srep28563 (PMC4935895; doi:10.1038/srep28563)
Supplement: Supplementary Information [file srep28563-s1.pdf]

# Optimal Perceived Timing: Integrating Sensory Information with Dynamically Updated Expectations

## Supplementary Information

Massimiliano Di Luca[1*] & Darren Rhodes[1]

[1]*Centre for Computational Neuroscience and Cognitive Robotics, School of Psychology, University of Birmingham, Edgbaston, Birmingham B15 2TT, UK.*

[*]m.diluca@bham.ac.uk

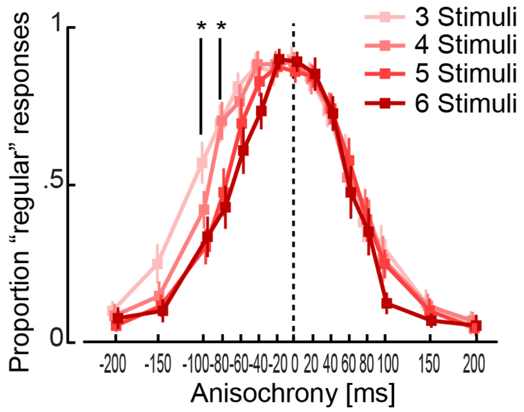


Supplementary Figure S1. Proportion of “regular” responses in Experiment 1S plotted as a function of the anisochrony of the last audio stimulus. Sequence lengths were presented in different blocks of trials (rather than interleaved as in Experiment 1). Fifteen new participants took part in this experiment. Asterisks denote asynchronies at which the proportion of responses differs across sequence lengths showing a change in responses for stimuli presented earlier than expected.

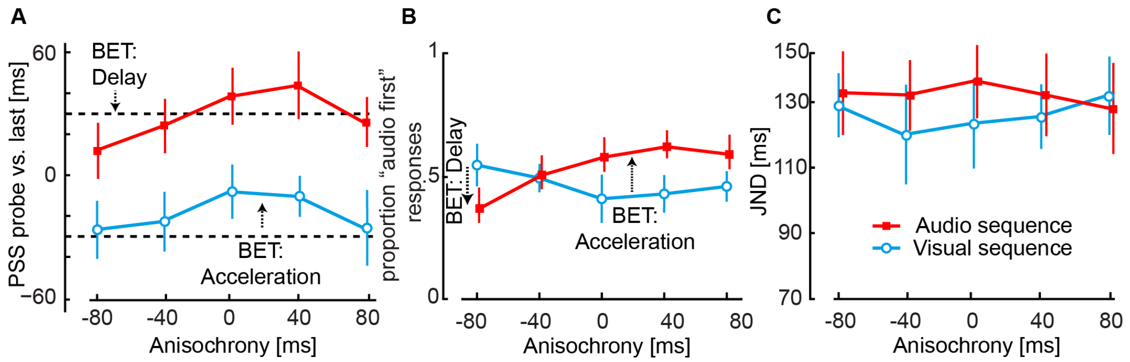


Supplementary Figure S2. Additional analysis of Experiment 2 data. (A) PSS values presented as the asynchrony between probe vs. last repeated stimulus (and not light vs. sound as depicted in Figure 3A and whose results are shown in Figure 2B). The dotted line represents a PSS of 30 ms between lights and sounds, which is reversed in sign for the visual sequence condition. The BET is evidenced as an equal distortion of the two curves. A two-way r.m. ANOVA with sequence modality and anisochrony as factors, evidences the difference in latency between audio and visual stimuli ($F(1,11)=7.1$, $p=.022$, $\eta p^2=.39$), as well as the BET effect (factor anisochrony, $F(4,44)=4.8$, $p=.0026$, $\eta p^2=.30$), but no interaction ($F(4,44)=.4$, $p=.80$). (B) Proportion of “audio first” responses for physically synchronous audio and visual stimuli. The BET is evidenced as the difference between the two curves. For example, in an isochronous auditory sequence with a physically synchronous light stimulus, there is an increase in audio-first responses, which evidences the auditory stimulus being accelerated. A two-way r.m. ANOVA with sequence modality and anisochrony as factors, yielded no main effects but a significant interaction $F(4,44)=4.6$, $p=.0033$, $\eta p^2=.30$. (C) JND values. A two-way r.m.

ANOVA with sequence modality and anisochrony as factors indicates that no main effects or interaction are significant (all $p > .3$).
